# Genetic Variation and Demographic History of Green Weevil *Hypomeces pulviger* (Herbst, 1795) (Coleoptera: Curculionidae) in Thailand Examined by Mitochondrial DNA Sequences

**DOI:** 10.3390/biology15161442

**Published:** 2026-08-21

**Authors:** Nakorn Pradit, Warayutt Pilap, Chavanut Jaroenchaiwattanachote, Jatupon Saijuntha, Wittaya Tawong, Watee Kongbuntad, Panida Laotongsan, Komgrit Wongpakam, Khamla Inkhavilay, Isara Thanee, Weerachai Saijuntha, Chairat Tantrawatpan

**Affiliations:** 1 Walai Rukhavej Botanical Research Institute, Mahasarakham University, Maha Sarakham 44150, Thailand; nakorn.p@msu.ac.th (N.P.); panida_walai@yahoo.com (P.L.); komwongpa@gmail.com (K.W.); 2 Center of Excellence in Biodiversity Research, Mahasarakham University, Maha Sarakham 44150, Thailand; chavanut.j@msu.ac.th (C.J.); isara.t@msu.ac.th (I.T.); weerachai.s@msu.ac.th (W.S.); 3 Biomedical Science Research Unit, Mahasarakham University, Maha Sarakham 44150, Thailand; 4 Faculty of Engineering, Mahasarakham University, Maha Sarakham 44150, Thailand; jatupons2534@gmail.com; 5 Department of Agricultural Sciences, Faculty of Agriculture Natural Resources and Environment, Naresuan University, Phitsanulok 65000, Thailand; wittayat@nu.ac.th; 6 Program in Biotechnology, Faculty of Science, Maejo University, Chiang Mai 50290, Thailand; watee@mju.ac.th; 7 Center of Excellence in Biodiversity, National University of Laos, Vientiane 7322, Laos; khamla.inkhavilay@nuol.edu.la; 8 Department of Biology, Faculty of Science, Mahasarakham University, Maha Sarakham 44150, Thailand; 9 Faculty of Medicine, Mahasarakham University, Maha Sarakham 44000, Thailand; 10 Division of Cell Biology, Department of Preclinical Sciences, Faculty of Medicine, and Center of Excellence in Stem Cell Research and Innovation, Thammasat University, Rangsit Campus, Pathum Thani 12120, Thailand

**Keywords:** edible insect, pest, population genetic, haplotype diversity, phylogeny

## Abstract

The population genetic diversity and demographic history of *Hypomeces pulviger* in Thailand were examined based on mitochondrial cytochrome c oxidase subunit 1 (*CO1*) and 16S ribosomal DNA (16S rDNA) sequence data. A total of 171 and 104 individuals from multiple populations were analyzed using *CO1* and 16S rDNA sequences, respectively. The *CO1* sequence dataset revealed high haplotype diversity (Hd = 0.999) and moderate nucleotide diversity (Nd = 0.0323), whereas the 16S rDNA showed lower diversity (Hd = 0.750, Nd = 0.0026). Population structure analyses showed low to moderate differentiation in *CO1* sequences with a significant isolation-by-distance pattern, suggesting distance-limited gene flow, while 16S rDNA sequences showed weaker structure. Neutrality tests and mismatch distribution analyses supported a recent population expansion, as indicated by significantly negative Fu’s *F*s and a unimodal distribution. Haplotype network and phylogenetic analyses further demonstrated greater resolution in the *CO1* gene compared to the *16S rRNA* gene. Collectively, *H. pulviger* populations in Thailand are genetically diverse, connected, and expanding, likely facilitated by both natural dispersal and agricultural activities. These findings provide important insights for understanding pest dynamics and developing effective management strategies.

## Simple Summary

The green weevil, *Hypomeces pulviger*, is a significant agricultural pest in Thailand. This study examined its genetic variation using mitochondrial genes (*CO1* and *16S rRNA*) to understand its population structure and dispersal. The results showed high genetic diversity and evidence of recent population expansion. Populations were generally connected, with gene flow occurring more frequently between nearby areas, although long-distance dispersal was also likely. These findings suggest that *H. pulviger* is actively spreading, likely facilitated by both natural movement and agricultural activities, with important implications for pest management and control.

## 1. Introduction

Insects play a pivotal role in agroecosystems, influencing both agricultural productivity and providing an alternative source of food as edible insects. While many insect species are regarded primarily as crop pests, others simultaneously contribute to human nutrition as edible insects [1]. Understanding the biological and genetic characteristics of such dual-role species is increasingly important for developing integrated strategies that balance pest management with sustainable food use [2]. The green weevil *Hypomeces pulviger* (Herbst, 1795) (Coleoptera: Curculionidae) represents a notable example of this complexity in Thailand. However, the taxonomy of this species remains controversial, and numerous synonyms have been reported in the literature, such as *Curculio orientalis* Olivier, 1807; *C. pulviger* Herbst, 1795; *C. squamosus* Fabricius, 1792; *Hypomeces auricephalus* Faust, 1893; *H. aurulentus* Herbst, 1797; *H. dispar* Faust, 1894; *H. fabricii* Faust, 1893; *H. gossypi* Matsumura, 1915; *H. peregrinus* Matsumura, 1915; *H. pulverulentus* Fabricius, 1792; and *H. squamosus* (Fabricius, 1792) [3].

*Hypomeces pulviger* is a polyphagous insect widely distributed throughout South and Southeast Asia and is recognized as an agricultural pest of economic concern. Adult weevils feed on foliage, causing defoliation, while larvae inhabit the soil and feed on plant roots, potentially impairing plant growth and yield [4]. In Thailand, this species has been reported to attack a wide range of economically important crops, including fruit trees such as durian and citrus, as well as field crops including sweet potato, legumes, and other cultivated plants. This broad host range enhances its potential to persist across agricultural landscapes, thereby increasing its economic importance [5]. Crop damage caused by *H. pulviger* can be particularly detrimental in smallholder and mixed farming systems, where pest outbreaks may result in reduced crop production and economic losses for farming communities. Effective management of this pest therefore requires sound understandings of its population structure, dispersal ability, and genetic diversity, which provide essential information for predicting gene flow, identifying invasion pathways, and designing sustainable pest management strategies [6].

At the same time, *H. pulviger* is traditionally consumed as an edible insect in several regions of Thailand, particularly in the northeastern region. Adult weevils are seasonally collected from the wild and consumed as part of the long-standing entomophagy traditions practiced in rural communities, where edible insects represent an important seasonal food resource [7]. Like many other edible insects consumed in Thailand, *H. pulviger* provides an alternative source of dietary protein and other essential nutrients [8]. More broadly, edible insects are increasingly recognized for their potential to contribute to sustainable food systems because of their high nutritional value, relatively low environmental footprint, and accessibility to local communities. In Thailand, where entomophagy is deeply rooted in local culture, edible insects such as *H. pulviger* can contribute to dietary diversity and nutritional resilience, particularly in rural areas.

Understanding the population genetic structure of pest species provides essential information for predicting dispersal, estimating gene flow, reconstructing demographic history, and assessing the evolutionary potential of populations. These processes directly influence the spread of adaptive traits, including insecticide resistance and host adaptation, and ultimately determine the effectiveness of regional pest management programs [9]. Recent studies on economically important weevils have demonstrated that genetic diversity and population structure are strongly influenced by dispersal ability, host distribution, and agricultural practices. For example, the Camellia weevil *Curculio chinensis* (Chevrolat, 1978) exhibits high mitochondrial diversity, significant isolation by distance, and evidence of recent demographic expansion, which are associated with host availability and landscape connectivity [10]. Similarly, population genetic studies of the banana corm weevil *Cosmopolites sordidus* (Germar, 1824) and *Ceutorhynchus neglectus* Blatchley, 1916 have revealed that extensive gene flow and human-mediated movement of host plants contribute substantially to population connectivity over large geographic areas [11,12]. These studies demonstrate how population genetic analyses provide valuable information for understanding pest dispersal and developing effective management strategies.

Mitochondrial DNA (mtDNA) markers are widely used to assess genetic variation and population structure in insect pests because of their relatively high mutation rates, maternal inheritance, and lack of recombination. These characteristics make mtDNA particularly useful for inferring population differentiation, gene flow, phylogeographic patterns, and demographic history [13]. Despite the agricultural importance and food-related relevance of *H. pulviger* in Thailand, information on its genetic variation remains limited. The present study, therefore, investigates mitochondrial DNA sequence variation in *H. pulviger* populations across Thailand, aiming to generate baseline genetic data that can inform pest management practices, while also contributing to discussions on sustainable use of insects as a food resource.

## 2. Materials and Methods

### 2.1. Field Collection of Specimens

**Figure 1:**
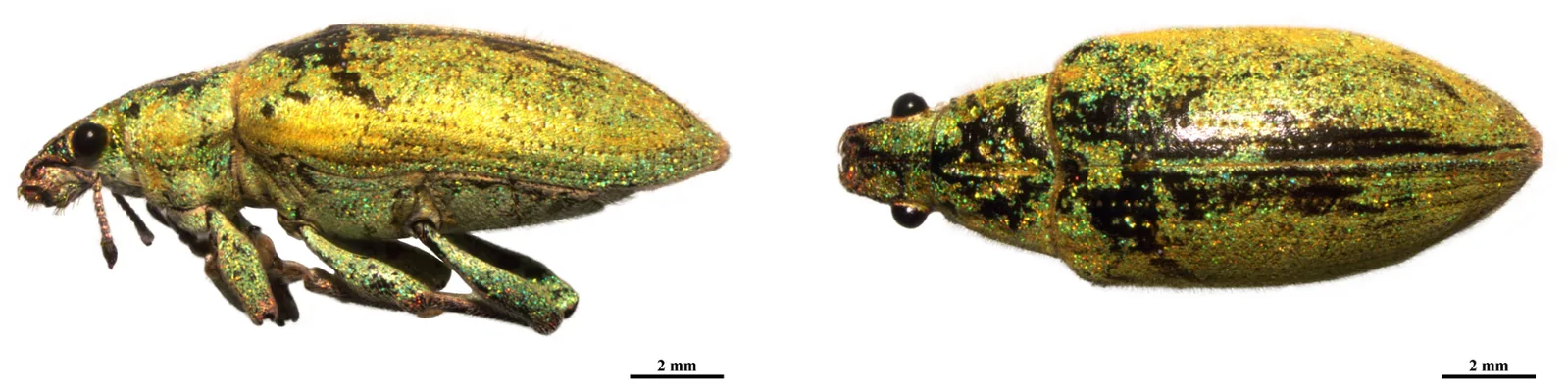
Adult of green weevil *Hypomeces pulviger* shown in lateral (**left**) and dorsal (**right**) views.

**Figure 2:**
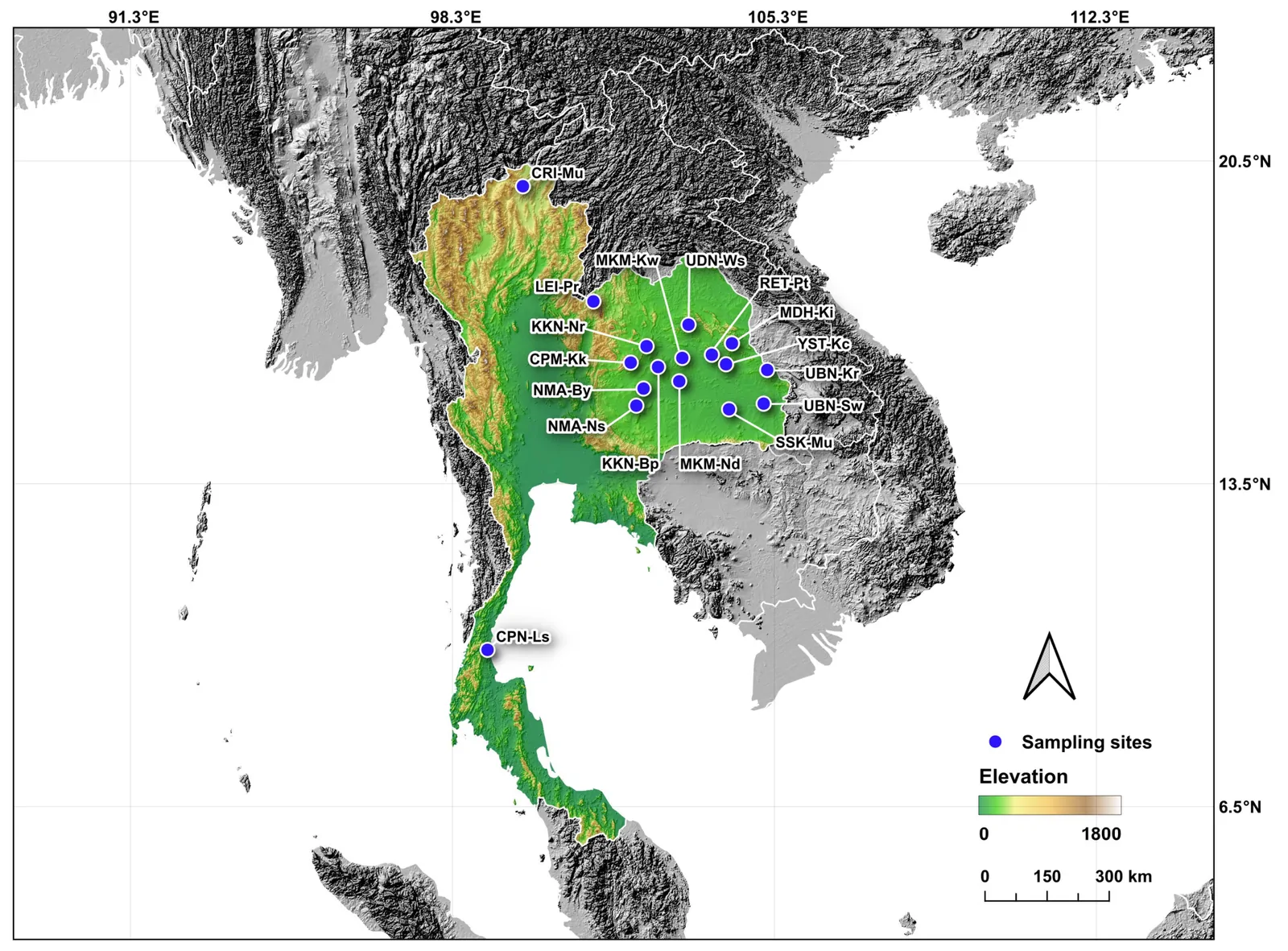
Map showing the geographical distribution of sampled *Hypomeces pulviger* across 17 localities in Thailand.

The 171 adults *H. pulviger* (Figure 1) were collected from leaves and branches of their host trees using a combination of sweep-netting and hand-picking from 17 different localities in Thailand (Figure 2 and Table 1). Adult specimens were immobilized by placing them on ice for approximately 10 min and were subsequently preserved in 80% ethanol. Species identification was performed based on morphological characteristics using a taxonomic key for the family Curculionidae [14].

### 2.2. Molecular Analysis

Genomic DNA was extracted individually from the femoral tissue of the left foreleg of each *H. pulviger* specimen using the E.Z.N.A.^®^ Tissue DNA Kit (Omega Bio-tek, Norcross, GA, USA) according to the manufacturer’s instructions. Extracted DNA was stored at −20 °C until molecular analyses were performed. Partial mitochondrial cytochrome *c* oxidase subunit 1 (*CO1*) and 16S ribosomal DNA (16S rDNA) fragments were amplified by PCR using the primer sets and thermal cycling conditions described by Pradit et al. [15]. Amplification products were separated by electrophoresis on 1% agarose gels and stained with GelRed™ Nucleic Acid Gel Stain (Biotium, Hayward, CA, USA) for visualization. Target PCR bands were excised from the gels and purified using the E.Z.N.A.^®^ Gel Extraction Kit (Omega Bio-tek, Norcross, GA, USA). Purified amplicons were subsequently submitted to ATGC Co., Ltd. (Khlong Luang, Pathum Thani, Thailand) for bidirectional DNA sequencing.

### 2.3. DNA Sequence Processing and Population Genetic Analyses

The *CO1* (635 bp) and 16S rDNA (490 bp) sequences generated in this study, together with homologous sequences obtained from GenBank, were aligned using the online implementation of MAFFT version 7 [16]. The resulting alignments were subsequently inspected and manually refined where necessary using BioEdit version 7.2.5 [17]. Genetic diversity indices and haplotype characteristics were estimated with DnaSP version 5 [18]. Median-joining haplotype networks were generated in PopART version 1.7 [19] following the method of Bandelt et al. [20] using all sequences obtained in the present study. Nucleotide polymorphism, genetic differentiation (*Φ*_ST_), neutrality tests, and mismatch distribution analyses were conducted using the Arlequin program version 3.5.2.2 [21]. Isolation-by-distance (IBD) patterns were evaluated using R version 4.5.0 [22] by conducting partial Mantel tests with 1000 permutations at both fine and broad spatial scales, as implemented in the ade4 package [23].

### 2.4. Phylogenetic Analyses

Phylogenetic relationships were inferred using Bayesian Inference (BI), Maximum Likelihood (ML), and Neighbor-Joining (NJ) approaches. The most appropriate nucleotide substitution models for the *CO1* and 16S rDNA datasets were selected based on the corrected Akaike Information Criterion (AICc) implemented in MrModeltest version 2.4. [24]. The corrected AIC identified GTR + I + G as the most suitable substitution model for *CO1* and GTR + G for 16S rDNA. These model parameters were incorporated into the subsequent ML and BI phylogenetic analyses. Bayesian Inference (BI) analyses were conducted in BEAST X version 10.5.0 [25] using 30 million Markov chain Monte Carlo (MCMC) generations, with trees sampled every 1000 generations. MCMC convergence and effective sample sizes (ESS > 200) were evaluated using TRACER version 1.7.2 [26]. The initial 10% of sampled trees from each run were discarded as burn-in before generating the consensus tree, which was subsequently visualized in FigTree version 1.4.4 [27]. Maximum Likelihood (ML) and Neighbor-Joining (NJ) analyses were conducted in MEGA12 [28]. The ML analysis employed the substitution models selected above, whereas the NJ analysis was based on the Kimura 2-parameter (K2P) model with 1000 bootstrap replicates to assess node support. Sequences of *H. pulviger* from previous studies were also included in the analysis, namely GenBank accession numbers MH686497 [29] and PV988010 [30], which originated from specimens collected in Chiang Mai and Chonburi provinces, Thailand, respectively.

## 3. Results

### 3.1. Genetic Diversity Analysis

Analysis of mitochondrial DNA sequences of *H. pulviger* across Thailand revealed substantial genetic variation based on 635 bp *CO1* and 490 bp 16S rDNA sequences. The *CO1* sequence dataset comprised 171 individuals from 17 populations and identified 159 polymorphic sites (S), defining 159 haplotypes (H), of which 153 were unique (Uh). Transitions (147 sites) greatly outnumbered transversions (40 sites). At the population level, the number of polymorphic sites ranged from 6 to 84, whereas the number of haplotypes ranged from 2 to 21. Haplotype diversity (Hd) was consistently high across populations, ranging from 0.867 ± 0.129 to 1.000, with an overall value of 0.999 ± 0.001. Nucleotide diversity (π) ranged from 0.0050 ± 0.0012 to 0.0378 ± 0.0189, with an overall mean of 0.0323 ± 0.0005 (Table 2).

All 635-bp *CO1* sequences of Thai *H. pulviger* (159 haplotypes) were successfully translated into 211 amino acid residues, with no stop codons detected. A total of nine amino acid positions (4.27%) were identified as polymorphic. Amino acid substitutions were observed at positions 1 (Valine, V → Methionine, M), 65 (Alanine, A → Proline, P), 90 (Valine, V → Isoleucine, I), 113 (Serine, S → Alanine, A), 115 (Valine, V → Glycine, G), 144 (Arginine, R → Glutamine, Q), 146 (Alanine, A → Valine, V), 157 (Alanine, A → Valine, V), and 161 (Methionine, M → Threonine, T or Lysine, K) (Table S1).

In contrast, the 16S rDNA sequence dataset included 104 individuals from 15 populations and identified 30 polymorphic sites, comprising 21 transitions and 9 transversions. These polymorphic sites defined 33 haplotypes, of which 25 were unique. The number of polymorphic sites per population ranged from 1 to 8, while the number of haplotypes ranged from 2 to 7. Haplotype diversity was moderate to high, ranging from 0.500 ± 0.265 to 1.000 ± 0.500, with an overall value of 0.750 ± 0.045. Nucleotide diversity (π) remained consistently low across populations, ranging from 0.0010 ± 0.0005 to 0.0045 ± 0.0012, with an overall mean of 0.0026 ± 0.0003 (Table 2).

### 3.2. Genetic Differences Analysis

**Figure 3:**
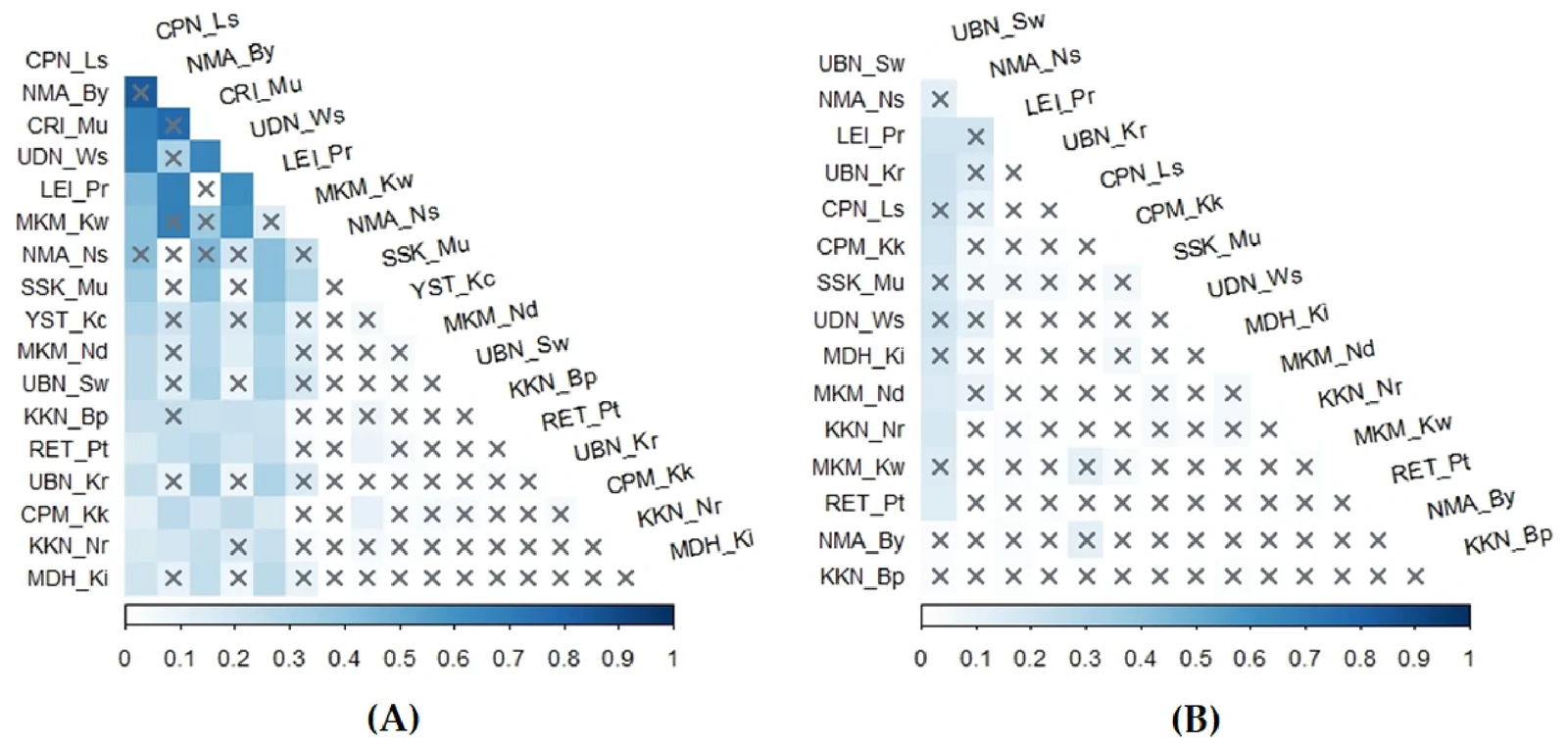
Heat maps illustrating pairwise genetic differentiation (*Φ*_ST_) among *Hypomeces pulviger* populations from Thailand based on mitochondrial *CO1* (**A**) and 16S rDNA (**B**) sequence data. Color intensity corresponds to *Φ*_ST_ values ranging from 0 to 1, with darker shades indicating greater genetic differentiation. Population codes are shown along the *y*-axis and correspond to the sampling localities listed in Table 1. Crosses (×) denote non-significant pairwise comparisons (*p* ≥ 0.05), whereas the values without a cross indicate significant genetic differentiation (*p* < 0.05).

Pairwise population differentiation further highlighted contrasting patterns between the *CO1* and 16S rDNA sequence datasets. The *CO1* sequence dataset exhibited relatively higher and more heterogeneous *Φ*_ST_ values among populations, reflecting its greater number of informative polymorphic sites and higher genetic resolution (Figure 3A). Despite this, many population pairs, espeically in the northeastern region showed low or non-significant differentiation. This is consistent with the high haplotype diversity and widespread distribution of unique haplotypes, suggesting that populations are not strongly isolated. Nevertheless, many population pairs, particularly those from northeastern Thailand, exhibited low or non-significant genetic differentiation, consistent with the high haplotype diversity and widespread distribution of unique haplotypes. In contrast, the CPN_Ls population from southern Thailand, the CRI_Mu population from northern Thailand, and the LEI_Pr population from northeastern Thailand showed significant genetic differentiation from many other populations. This geographic pattern was consistent with IBD, whereby genetic differentiation increases with spatial separation. In comparison, the 16S rDNA sequence dataset showed generally low to moderate *Φ*_ST_ values among most populations, with many pairwise comparisons indicating weak structure, likely reflecting the more conserved nature of this marker and its lower resolution for detecting fine-scale genetic differentiation (Figure 3B).

**Figure 4:**
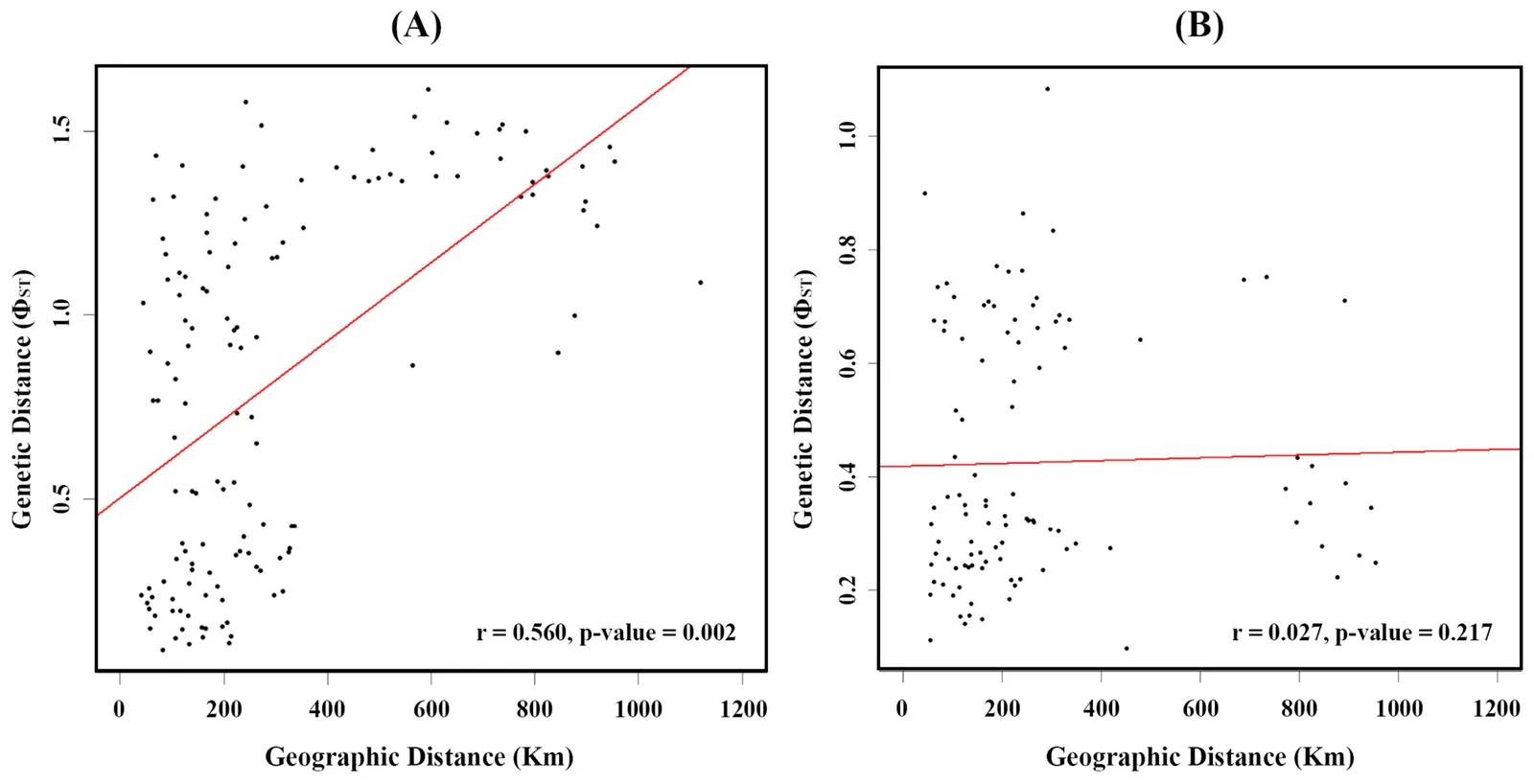
Mantel test plots illustrating isolation by distance (IBD) among *Hypomeces pulviger* populations based on *CO1* (**A**) and 16S rDNA (**B**) sequences. Pairwise *Φ*_ST_ values are plotted against Euclidean geographic distance (km). r denotes the correlation coefficient, and *p* indicates the corresponding significance level.

Isolation by distance analyses revealed contrasting spatial genetic patterns between the two mitochondrial markers. The *CO1* sequence dataset exhibited a significant moderate positive correlation between genetic and geographic distance (r = 0.56, *p*-value = 0.002), indicating a clear IBD pattern (Figure 4A). This suggests that genetic differentiation increases with geographic distance. Despite the high haplotype diversity observed in *CO1*, this pattern indicates that dispersal is not entirely random and that geographic distance still plays a role in structuring populations, possibly due to limitations in natural dispersal or localized adaptation. In contrast, the 16S rDNA sequence dataset showed no significant correlation between genetic and geographic distance (r = 0.027, *p*-value = 0.217), indicating the absence of a detectable IBD pattern (Figure 4B). This suggests that genetic differentiation in 16S rDNA is not strongly influenced by geographic distance, likely due to its lower mutation rate and reduced resolution, which limits its ability to capture fine-scale spatial structure.

### 3.3. Demographic History and Neutrality Tests

**Figure 5:**
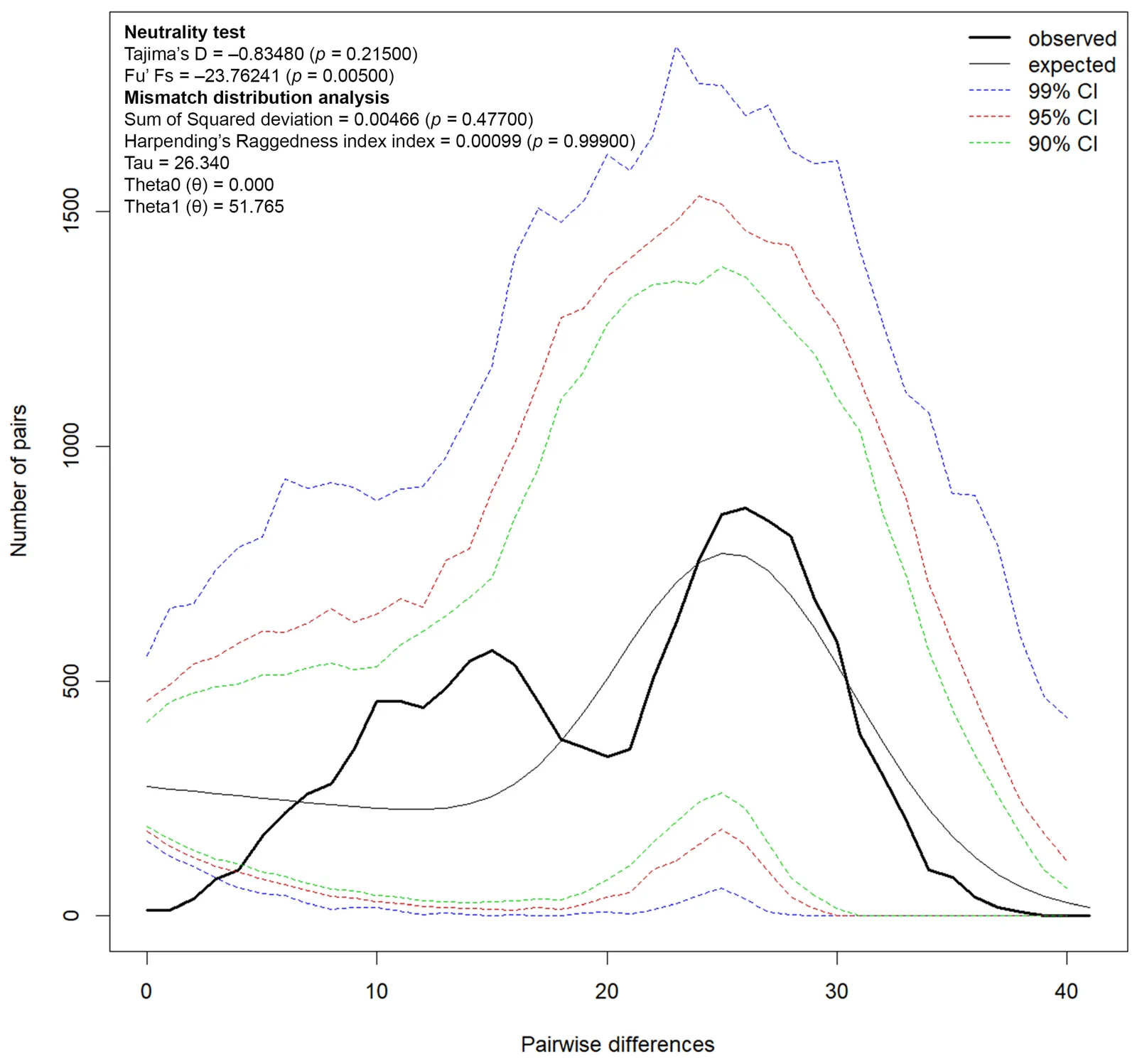
Mismatch distribution analysis and neutrality test for *Hypomeces pulviger* inferred from *CO1* sequence data. The thick solid curve depicts the observed distribution of pairwise nucleotide differences, and the thin solid curve represents the expected distribution assuming a sudden demographic expansion.

Based on the *CO1* sequences, neutrality tests and mismatch distribution analyses (Figure 5) indicated a negative Tajima’s *D* value that was not statistically significant (*D* = −0.83480, *p* = 0.21500). In contrast, Fu’s *F*s was significantly negative (*F*s = −23.76241, *p* = 0.00500). The observed mismatch distribution closely approximated the expected distribution predicted by a sudden population expansion model, exhibiting a unimodal profile with a peak at intermediate pairwise differences. Goodness-of-fit statistics indicated no significant deviation from the expansion model, as evidenced by the non-significant sum of squared deviations (SSD = 0.00466, *p* = 0.47700) and Harpending’s raggedness index (r = 0.00099, *p* = 0.99900). The estimated demographic parameters included τ = 26.340, θ_0_ = 0.000, and θ_1_ = 51.765, with an effective population size estimated at approximately Ne = 2.26 × 10^6^.

### 3.4. Haplotype Network and Phylogenetic Tree Analyses

**Figure 6:**
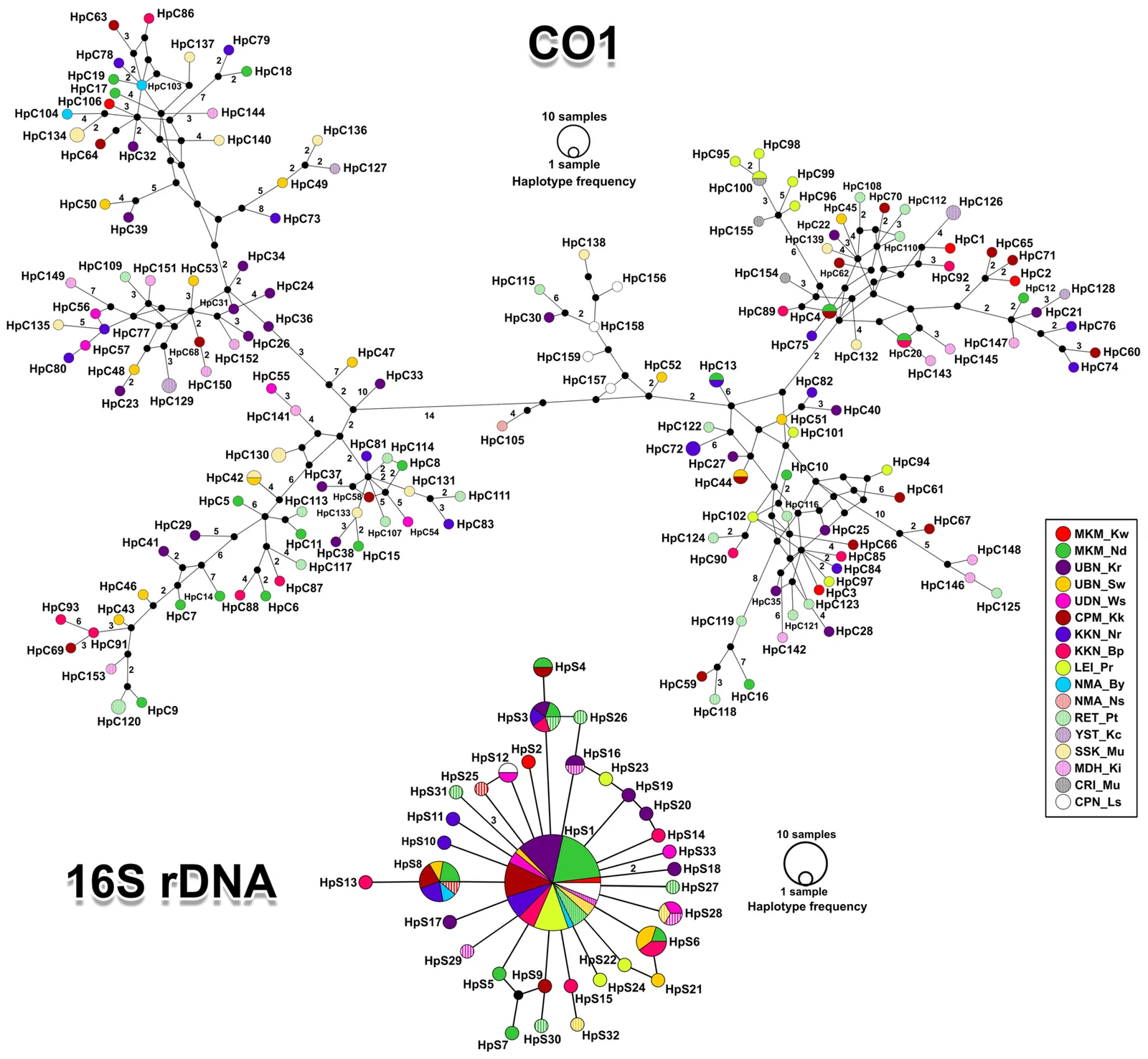
Haplotype networks of *Hypomeces pulviger* populations in Thailand inferred from mitochondrial *CO1* and 16S rDNA sequences. Circle colors represent the geographic regions of the sampling localities (see Table 1), and circle size is proportional to the number of individuals sharing each haplotype. Numbers along the connecting branches indicate the number of mutational steps when greater than one.

Haplotype network analysis revealed contrasting patterns between the *CO1* and 16S rDNA sequence datasets (Figure 6). The *CO1* haplotype network showed a highly complex structure comprising numerous haplotypes connected by multiple mutational steps, with many unique and low-frequency haplotypes distributed across populations. The network was characterized by several loosely connected clusters and long branches, indicating substantial haplotype divergence and a high number of mutational differences among haplotypes. In contrast, the 16S rDNA haplotype network exhibited a simpler structure with fewer haplotypes and a more centralized configuration. A dominant haplotype was observed at the center of the network, from which several low-frequency haplotypes radiated, forming a star-like pattern with relatively few mutational steps. Haplotype sharing among populations was more evident in the 16S rDNA sequence dataset than in *CO1*, reflecting lower genetic variation and reduced haplotype differentiation.

Phylogenetic analyses based on mitochondrial *CO1* and 16S rDNA sequences revealed distinct patterns of genetic relationships among *H. pulviger* haplotypes. The *CO1* phylogeny (Figure S1) showed a highly resolved topology with numerous haplotypes forming multiple subclades, although most internal nodes were supported by low to moderate bootstrap and posterior probability values. The two *CO1* sequences of *H. pulviger* previously reported from Chiang Mai (MH686497) and Chonburi (PV988010) provinces, Thailand, clustered within the *H. pulviger* clade together with the sequences obtained in the present study. The tree exhibited a relatively complex branching pattern with short internal branches and limited deep divergence, indicating shallow genetic structuring among haplotypes. In contrast, the 16S rDNA phylogeny (Figure S2) displayed a simpler topology with fewer haplotypes and more compact clustering. Several clades were moderately to strongly supported, including one well-supported lineage (e.g., bootstrap/posterior probability = 99/100), while most other nodes showed moderate support. A previously reported 16S rDNA sequence of *H. pulviger* from Chonburi province (PV988010), clustered within the *H. pulviger* clade together with all sequences obtained in the present study. For both markers, all haplotypes of *H. pulviger* formed a monophyletic group clearly separated from the outgroup taxa (*Niphades castanea* Chao, *Sympiezomias velatus* (Chevrolat, 1845), *Propomacrus davidi* Deyrolle, 1874, and *Copris tripartitus* Waterhouse, 1875).

## 4. Discussion

The present study revealed substantial genetic diversity in *H. pulviger* populations across Thailand, with contrasting patterns between the *CO1* and 16S rDNA markers. The *CO1* dataset exhibited extremely high haplotype diversity together with moderate nucleotide diversity, a genetic signature commonly associated with rapid population expansion following historical bottlenecks or founder events [31]. In contrast, the 16S rDNA marker displayed considerably lower haplotype and nucleotide diversity, reflecting its more conserved nature and slower evolutionary rate [32]. These contrasting patterns demonstrate the complementary roles of the two mitochondrial markers. The rapidly evolving *CO1* marker provided greater resolution for detecting recent demographic processes and fine-scale population differentiation, whereas the more conserved 16S rDNA marker better represented deeper evolutionary relationships. The use of both markers therefore provides a more comprehensive understanding of the evolutionary history and contemporary population dynamics of *H. pulviger* than could be achieved using either marker alone.

The high genetic diversity observed in *H. pulviger* shows considerable evolutionary potential that may enhance its ability to adapt to changing environmental conditions, overcome host plant defenses, and respond to pest management practices [33,34]. This adaptive potential is particularly important because *H. pulviger* is a polyphagous pest that attacks numerous economically important crops, including longan, lychee, durian, mango, citrus, and several other cultivated plants [4,35]. The availability of diverse host plants may promote genetic diversification by providing heterogeneous ecological niches that facilitate local adaptation and host-associated differentiation [36,37]. Although the present study was not specifically designed to evaluate host-associated genetic structure, the exceptionally large number of unique *CO1* haplotypes raises the possibility that populations associated with different host plants may be undergoing ecological divergence. Similar host-associated genetic lineages have been documented in several phytophagous insects, including the apple maggot fly *Rhagoletis pomonella* (Walsh, 1867) and the pea aphid *Acyrthosiphon pisum* Harris, 1776, in which sympatric populations diverged despite extensive geographic overlap [38,39]. Future studies incorporating balanced sampling across different host plants and nuclear genetic markers will be necessary to evaluate this hypothesis.

Population genetic analyses further demonstrated contrasting patterns between the two mitochondrial markers. The *CO1* dataset revealed relatively heterogeneous but generally low to moderate pairwise genetic differentiation (*Φ*_ST_), indicating substantial gene flow among populations. Furthermore, the significant IBD pattern showed that genetic differentiation increased progressively with geographic distance, consistent with restricted dispersal among populations [40]. In contrast, the 16S rDNA dataset exhibited weaker genetic structure and no detectable IBD pattern, most likely because of its lower mutation rate and reduced number of informative polymorphic sites [41]. Similar differences between rapidly and slowly evolving mitochondrial markers have been widely reported in insects, where *CO1* often provides greater resolution to detect recent population processes than more conserved mitochondrial genes [42,43].

The haplotype networks and phylogenetic analyses strongly supported these findings. The *CO1* haplotype network consisted of numerous unique haplotypes connected by multiple mutational steps, reflecting extensive genetic variation accumulated during recent population diversification. In contrast, the 16S rDNA network exhibited a simpler and more centralized topology with extensive haplotype sharing among populations, indicating lower genetic variability. Likewise, the shallow branching pattern observed in the *CO1* phylogeny, together with generally low internal support despite numerous haplotypes, suggests that most genetic variation accumulated relatively recently and has not yet resulted in deep evolutionary divergence. Such shallow genealogical structure accompanied by high haplotype diversity is characteristic of populations that have undergone rapid demographic expansion while maintaining substantial contemporary gene flow [44].

The coexistence of significant IBD and high haplotype diversity suggests that geographic distance has influenced the distribution of mitochondrial genetic variation. Although mtDNA data do not directly measure dispersal or gene flow, the observed genetic patterns are consistent with predominantly local dispersal, with occasional long-distance movements potentially associated with human-mediated transport, such as the movement of nursery stock, seedlings, and other agricultural materials. Similar patterns have been reported in other economically important weevils, including *C. chinensis*, *C. sordidus*, and *C. neglectus*, where both natural dispersal and human-mediated transport strongly influence population connectivity over broad geographic scales [10,11,12]. Consequently, effective management of *H. pulviger* should be based on both local dispersal and anthropogenic movement when designing regional monitoring and control strategies.

Demographic analyses further suggested the recent population expansion of *H. pulviger* throughout Thailand. The significantly negative Fu’s *F*s statistic, together with the unimodal mismatch distribution and the non-significant sum of squared deviations (SSD) and Harpending’s raggedness index, are consistent with the expectations of a sudden demographic expansion model [45,46]. Although Tajima’s *D* was negative but not statistically significant, Fu’s *F*s is generally considered more sensitive for detecting recent demographic expansion, particularly in datasets characterized by high haplotype diversity [47]. These findings, together with the star-like haplotype network, are consistent with a scenario of recent population growth and range expansion of *H. pulviger*. Similar demographic expansion has been documented in numerous Southeast Asian insects and is frequently attributed to climatic oscillations during the late Pleistocene, which repeatedly altered vegetation, host plant distributions, and habitat connectivity [48,49,50]. Although the precise timing of expansion cannot be estimated because the generation time of *H. pulviger* remains unknown, historical climatic changes are likely to have contributed to shaping its present genetic structure. However, these demographic inferences should be interpreted with caution because they are based solely on mitochondrial DNA, and alternative explanations, such as selective sweeps or other demographic processes, cannot be excluded.

A limitation of the present study is that population genetic inference was based exclusively on mitochondrial DNA markers. Although mitochondrial genes are highly informative for investigating maternal lineages, demographic history, and recent dispersal, they represent only a single maternally inherited locus and therefore may not fully capture genome-wide population processes. Incorporating biparentally inherited nuclear markers, such as microsatellites, SNPs, or genome-wide sequencing approaches, would provide a more comprehensive understanding of gene flow, admixture, and contemporary population connectivity. In addition, although specimens were collected from several host plant species, the sample sizes for individual hosts were insufficient to evaluate host-associated population differentiation. Future studies integrating broader geographic sampling, balanced host-plant representation, and genomic approaches will provide a more complete understanding of the evolutionary ecology of *H. pulviger*.

The present findings have important implications for sustainable pest management. The high genetic diversity, substantial gene flow, and evidence of recent demographic expansion showed that *H. pulviger* possesses considerable adaptive potential that may facilitate rapid responses to environmental change and pest control measures. Regional pest management programs should therefore emphasize continuous genetic monitoring, coordinated surveillance among agricultural regions, and careful management of plant movement to reduce long-distance dispersal. Because *H. pulviger* also serves as a traditionally consumed edible insect in parts of Thailand, understanding its population genetic structure contributes not only to agricultural pest management but also to the sustainable utilization and conservation of this valuable biological resource. Integrating molecular population genetics with ecological monitoring will therefore support both effective crop protection and sustainable management of edible insect resources in Thailand.

## 5. Conclusions

Collectively, this study provides the first nationwide assessment of the genetic diversity and population structure of *H. pulviger* in Thailand. The results revealed high genetic diversity and a pattern of genetic differentiation associated with geographic distance, while demographic analyses suggest a history of population expansion. These findings provide an important foundation to understand the population dynamics and dispersal patterns of this economically important pest and may contribute to the development of sustainable management strategies, as well as future studies on its utilization as an edible insect resource.

## Figures and Tables

**Table 1:** Collection localities and sampling information for *Hypomeces pulviger* populations from Thailand.

Code	Province	District	Analyzed Samples (*n*)		Coordinates
			*CO1*	16S rDNA	
MKM-Kw	Maha Sarakham ^a^	Kantharawichai	3	2	16.231° N/103.297° E
MKM-Nd	Maha Sarakham ^a^	Na Dun	17	17	15.724° N/103.231° E
UBN-Kr	Ubon Ratchathani ^b^	Khemmarat	21	14	15.971° N/105.144° E
UBN-Sw	Ubon Ratchathani ^a^	Sawang Wirawong	12	5	15.239° N/105.066° E
UDN-Ws	Udon Thani ^a^	Wang Sam Mo	4	4	16.952° N/103.433° E
CPM-Kk	Chaiyaphum ^a^	Kaeng Khro	16	10	16.129° N/102.177° E
KKN-Nr	Khon Kaen ^a^	Nong Ruea	15	9	16.486° N/102.520° E
KKN-Bp	Khon Kaen ^a^	Ban Phai	10	10	16.034° N/102.769° E
LEI-Pr	Loei ^d^	Phu Ruea	9	9	17.456° N/101.365° E
NMA-By	Nakhon Ratchasima ^a^	Bua Yai	2	2	15.567° N/102.455° E
NMA-Ns	Nakhon Ratchasima ^a^	Nong Sung	2	2	15.195° N/102.298° E
RET-Pt	Roi Et ^c^	Phon Thong	20	8	16.300° N/103.940° E
YST-Kc	Yasothon ^b^	Kut Chum	6	N/A	16.093° N/104.248° E
SSK-Mu	Sisaket ^a^	Mueang	14	4	15.117° N/104.314° E
MDH-Ki	Mukdahan ^c^	Khamcha-i	13	4	16.547° N/104.377° E
CRI-Mu	Chiang Rai ^d^	Mueang	3	N/A	19.954° N/99.831° E
CPN-Ls	Chumphon ^e^	Lang Suan	4	4	9.900° N/99.062° E
Total			171	104	

Host tree: ^a^ *Cassia fistula* L., ^b^ *Schleichera oleosa* (Lour.) Merr., ^c^ *Mangifera indica* L., ^d^ *Dimocarpus longan* Lour, ^e^ *Mitragyna speciosa* Korth. *n*: sample number; N/A: not analyzed.

**Table 2:** Genetic diversity indices of *Hypomeces pulviger* populations from 17 different geographic regions of Thailand based on mitochondrial *CO1* and 16S rDNA sequence data.

Populations	*CO1*						16S rDNA					
	*n*	S	H	Uh	Hd ± SD	Nd ± SD	*n*	S	H	Uh	Hd ± SD	Nd ± SD
MKM-Kw	3	14	3	3	1.000 ± 0.272	0.0147 ± 0.0040	2	1	2	1	1.000 ± 0.500	0.0020 ± 0.0010
MKM-Nd	17	74	17	14	1.000 ± 0.020	0.0327 ± 0.0016	17	7	7	2	0.662 ± 0.126	0.0023 ± 0.0007
UBN-Kr	21	84	21	21	1.000 ± 0.015	0.0312 ± 0.0017	14	7	7	4	0.692 ± 0.137	0.0023 ± 0.0007
UBN-Sw	12	59	12	10	1.000 ± 0.034	0.0321 ± 0.0021	5	3	4	1	0.900 ± 0.161	0.0029 ± 0.0008
UDN-Ws	4	21	4	4	1.000 ± 0.177	0.0189 ± 0.0045	4	2	3	1	0.833 ± 0.222	0.0020 ± 0.0007
CPM-Kk	16	75	16	14	1.000 ± 0.022	0.0313 ± 0.0028	10	4	4	1	0.644 ± 0.152	0.0020 ± 0.0007
KKN-Nr	15	66	14	13	0.990 ± 0.028	0.0327 ± 0.0021	9	4	5	2	0.806 ± 0.120	0.0022 ± 0.0005
KKN-Bp	10	54	10	9	1.000 ± 0.045	0.0325 ± 0.0034	10	7	7	3	0.911 ± 0.077	0.0032 ± 0.0006
LEI-Pr	9	25	9	8	1.000 ± 0.052	0.0161 ± 0.0017	9	4	4	3	0.583 ± 0.183	0.0018 ± 0.0007
NMA-By	2	6	2	2	1.000 ± 0.500	0.0095 ± 0.0047	2	1	2	0	1.000 ± 0.500	0.0020 ± 0.0010
NMA-Ns	2	24	2	2	1.000 ± 0.500	0.0378 ± 0.0189	2	2	2	1	1.000 ± 0.500	0.0041 ± 0.0020
RET-Pt	20	72	19	19	0.995 ± 0.018	0.0314 ± 0.0019	8	8	6	4	0.893 ± 0.111	0.0045 ± 0.0012
YST-Kc	6	44	4	4	0.867 ± 0.129	0.0347 ± 0.0055	n/a	n/a	n/a	n/a	n/a	n/a
SSK-Mu	14	60	12	11	0.978 ± 0.035	0.0279 ± 0.0031	4	3	3	1	0.833 ± 0.222	0.0031 ± 0.0011
MDH-Ki	13	71	13	13	1.000 ± 0.030	0.0367 ± 0.0023	4	3	4	1	1.000 ± 0.177	0.0031 ± 0.0007
CRI-Mu	3	11	3	2	1.000 ± 0.272	0.0116 ± 0.0037	n/a	n/a	n/a	n/a	n/a	n/a
CPN-Ls	4	6	4	4	1.000 ± 0.177	0.0050 ± 0.0012	4	1	2	0	0.500 ± 0.265	0.0010 ± 0.0005
Total	171	159	159	153	0.999 ± 0.001	0.0323 ± 0.0005	104	30	33	25	0.750 ± 0.045	0.0026 ± 0.0003

n/a = not analyzed.

## Data Availability

The sequences generated in this study have been deposited in GenBank under accession numbers PZ776235–PZ776267 for the 16S rDNA sequences and PZ778390–PZ778548 for the *CO1* sequences.

## Supplementary Materials

The following supporting information can be downloaded at https://www.mdpi.com/article/10.3390/biology15161442/s1. Table S1: Comparison of amino acid sequences among *CO1* haplotypes of *Hypomeces pulviger* examined in this study, including two sequences retrieved from GenBank. Figure S1: Maximum Likelihood phylogenetic tree inferred from *CO1* sequences of *Hypomeces pulviger*. Node support values represent Maximum Likelihood bootstrap (ML), Neighbor-Joining bootstrap (NJ), and Bayesian posterior probabilities (BI), and are shown above or beside the corresponding branches in the order ML/BI/NJ. The scale bar indicates 0.1 substitutions per nucleotide site. Taxa marked with * and ** indicate sequences retrieved from GenBank based on previous publications [29] and [30], respectively. The gray-shaded area denotes the outgroup taxa. Figure S2: Maximum Likelihood phylogenetic tree inferred from 16S rDNA sequences of *Hypomeces pulviger*. Node support values represent Maximum Likelihood bootstrap (ML), Neighbor-Joining bootstrap (NJ), and posterior probabilities for Bayesian Inference (BI), and are shown above or beside the corresponding branches in the order ML/BI/NJ. The scale bar indicates 0.05 substitutions per nucleotide site. The taxon marked with ** indicates a sequence retrieved from GenBank based on a previous publication [30]. The gray-shaded area denotes the outgroup taxa.
